# Protein kinase A-dependent Neuronal Nitric Oxide Synthase Activation Mediates the Enhancement of Baroreflex Response by Adrenomedullin in the Nucleus Tractus Solitarii of Rats

**DOI:** 10.1186/1423-0127-18-32

**Published:** 2011-05-19

**Authors:** David HT Yen, Lih-Chi Chen, Yuh-Chiang Shen, Ying-Chen Chiu, I-Chun Ho, Ya-Jou Lou, I-Chin Chen, Jiin-Cherng Yen

**Affiliations:** 1 Institute of Emergency and Critical Care Medicine, School of Medicine, National Yang-Ming University, Taipei, Taiwan; 2 Emergency Department, Taipei Veterans General Hospital, Taipei, Taiwan; 3 Department of Pharmacy, Taipei City Hospital, Taipei, Taiwan; 4 National Research Institute of Chinese Medicine, Taipei, Taiwan; 5 Institute of Pharmacology, School of Medicine, National Yang-Ming University, Taipei, Taiwan

## Abstract

**Background:**

Adrenomedullin (ADM) exerts its biological functions through the receptor-mediated enzymatic mechanisms that involve protein kinase A (PKA), or neuronal nitric oxide synthase (nNOS). We previously demonstrated that the receptor-mediated cAMP/PKA pathway involves in ADM-enhanced baroreceptor reflex (BRR) response. It remains unclear whether ADM may enhance BRR response via activation of nNOS-dependent mechanism in the nucleus tractus solitarii (NTS).

**Methods:**

Intravenous injection of phenylephrine was administered to evoke the BRR before and at 10, 30, and 60 min after microinjection of the test agents into NTS of Sprague-Dawley rats. Western blotting analysis was used to measure the level and phosphorylation of proteins that involved in BRR-enhancing effects of ADM (0.2 pmol) in NTS. The colocalization of PKA and nNOS was examined by immunohistochemical staining and observed with a laser confocal microscope.

**Results:**

We found that ADM-induced enhancement of BRR response was blunted by microinjection of NPLA or Rp-8-Br-cGMP, a selective inhibitor of nNOS or protein kinase G (PKG) respectively, into NTS. Western blot analysis further revealed that ADM induced an increase in the protein level of PKG-I which could be attenuated by co-microinjection with the ADM receptor antagonist ADM_22-52 _or NPLA. Moreover, we observed an increase in phosphorylation at Ser1416 of nNOS at 10, 30, and 60 min after intra-NTS administration of ADM. As such, nNOS/PKG signaling may also account for the enhancing effect of ADM on BRR response. Interestingly, biochemical evidence further showed that ADM-induced increase of nNOS phosphorylation was prevented by co-microinjection with Rp-8-Br-cAMP, a PKA inhibitor. The possibility of PKA-dependent nNOS activation was substantiated by immunohistochemical demonstration of co-localization of PKA and nNOS in putative NTS neurons.

**Conclusions:**

The novel finding of this study is that the signal transduction cascade that underlies the enhancement of BRR response by ADM in NTS is composed sequentially of cAMP/PKA and nNOS/PKG pathways.

## Background

Adrenomedullin (ADM), a 52-amino acid peptide, was originally isolated from human pheochromocytoma and initially shown to have potent vasodilatory activity [1]. The physiologic and pharmacologic functions of ADM have been intensively investigated after its discovery (for review see [2]). ADM exerts multiple biological activities by acting on its specific receptors, composed of calcitonin receptor-like receptor (CRLR) and receptor activity modifying protein (RAMP)-2 or -3 [3]. The hypotensive effect of intravenously administered ADM has been attributed to activation of ADM receptors (ADMRs) located on blood vessels [1]. In addition to distribution in the cardiovascular system, ADM and ADMRs are also expressed in the central nervous system (CNS) and are particularly localized to the autonomic nuclei, including nucleus tractus solitarii (NTS), lateral parabrachial nucleus (LPBN), and rostral ventrolateral medulla (RVLM) [4-6]. These findings suggested a possible role for ADM in central regulation of cardiovascular functions. Indeed, several studies demonstrated that microinjection of ADM into the CNS induces brain area-specific changes in arterial pressure and heart rate (HR) [7,8]. Other studies further indicated that central ADM also exhibits an area-specific regulation on the baroreceptor reflex (BRR) in anesthetized or conscious animals [9-11]. In our recent study [12], we demonstrated that microinjection of ADM into NTS, the termination site of primary baroreceptor afferents in the brain stem [13], significantly increases BRR response and sensitivity in a time- and dose-dependent manner, without producing discernible changes in basal arterial pressure and heart rate.

Stimulation of cyclic adenosine monophosphate (cAMP) formation is suggested to be the primary downstream mechanism subsequent to activation of the G_s _protein-coupled ADMRs in vascular cells [1,14]. In CNS neurons, the cAMP-associated mechanism is also considered to be the primary signaling pathway that mediates ADM actions. Xu and Krukoff reported that ADM inhibits the baroreflex control of HR via activation of cAMP-dependent protein kinase A (PKA) in RVLM of the rat [11]. Our previous study also revealed the involvement of cAMP/PKA-dependent mechanism in BRR augmentation in response to activation of ADMRs in NTS [12]. In addition to cAMP/PKA pathway, nitric oxide (NO) has been suggested to serve as another intracellular signaling molecule that mediates the ADM actions [2]. In RVLM and LPBN, ADM induces hypertensive effect through cyclic guanosine monophosphate (cGMP)-associated signaling that is mediated by NO derived from neuronal NO synthase (nNOS) [15,16]. However, whether the nNOS-dependent mechanism contributes to the BRR-enhancing effect of ADM in NTS remains unclear.

The present study was undertaken to evaluate the hypothesis that ADM may enhance BRR through PKA-dependent activation of nNOS in NTS. Our results support this hypothesis and reveal that nNOS may mediate ADM-induced BRR enhancement via activation of cGMP-dependent protein kinase G (PKG) in NTS. We further found that a PKA-dependent phosphorylation at the amino acid residue Ser1416 accounts for the ADM-induced nNOS activation.

## Materials and methods

### Animals

Sprague-Dawley rats (male, weighing 300-400 g) obtained from the Animal Center of National Yang-Ming University were used in this study. Rats were housed in a laboratory animal room under controlled temperature (25°C) and light on 0800-2000 h, and had unrestricted access to food and water. All animals were allowed to acclimatize for at least 3 days before use. Animal care and all experimental protocols applied in the present study were approved by the Institutional Animal Care and Use Committee of National Yang-Ming University.

### Surgical preparation

As described previously [12], rats were anesthetized by intraperitoneal (i.p.) injection of pentobarbital sodium (50 mg/kg) and placed on a heating pad. The trachea was intubated to facilitate ventilation, and the femoral artery was cannulated for monitoring systemic arterial pressure (SAP). The femoral veins on both sides were also cannulated for injection of test agents and administration of supplemental anesthetics. Mean AP (MAP, mmHg) and HR (beats/min) were derived from the pulsatile SAP signals measured with a pressure transducer (T844, ADInstruments, Castle Hill, Australia). To provide satisfactory anesthetic maintainance [17], rats received continuous infusion of pentobarbital at a rate of 15-20 mg/kg/h throughout the recording session.

### Microinjection

The rat was placed in a stereotaxic frame (Kopf, Tujunga, CA, USA) followed by an occipital craniotomy to expose the dorsal surface of the medulla. A glass pipette adapted to a Hamilton microsyringe (Reno, NV, USA) was used to microinject test agents into NTS. The coordinates used were: 0.5 mm rostral to the calamus scriptorius, ±0.5 mm lateral to the midline, and 0.5 mm below the surface of the medulla. The volume of injection was limited to 20 nl per site. For histological verification of injection sites, the microinjection medium for test agents or artificial cerebrospinal fluid (aCSF) contains 1% Evans blue.

### Test agents

ADM was purchased from Bachem AG (Hauptstrasse, Bubendorf, Switzerland); N-propyl-L-arginine (NPLA), S-methylisothiourea (SMT), L-NIO or 8-bromo-cAMP (8-Br-cAMP) from Tocris (Bristol, UK); Rp-8-bromo-cAMP (Rp-8-Br-cAMP) or Rp-8-bromo-cGMP (Rp-8-Br-cGMP) from Calbiochem (San Diego, CA, USA); ADM_22-52_, 3-morpholinosyndnomine (SIN-1), S-nitrosoglutathione (GSNO) or phenylephrine from Sigma-Aldrich (St. Louis, MO, USA); and L-NAME from Cayman (Ann Arbor, MI, USA).

### Measurement of BRR response

The procedures and methods for measuring the BRR response were described previously [12]. In brief, a bolus intravenous injection of phenylephrine (10 μg/kg) was administered to evoke the BRR before and 10, 30 or 60 min after microinjection of the test agent into NTS. The BRR response was represented by the ratio of the peak magnitude of reflex bradycardia to the peak magnitude of phenylephrine-induced pressor response. The averaged value of BRR response obtained from three injections of phenylephrine prior to microinjection of the test agent served as the baseline control.

### Histology

At the end of the physiological experiments, animals were killed with a high dose of pentobarbital sodium (100 mg/kg, i.p.). The brain stem was then removed and fixed in 10% paraformaldehyde-saline solution that contains 30% sucrose for 48-72 h. Serial sections were cut (20 μm) in a cryostat (Leica, Wetzlar, Germany) and mounted on slides. The sections were then stained with neutral red, and the microinjection site (marked with Evans blue) was identified under a microscope.

### Immunofluorescence staining

The procedures of triple immunofluorescence staining were described in a previous study [12]. Briefly, rats were deeply anesthetized and perfused transcardially with warm heparinized saline, followed by ice cold 4% paraformaldehyde (pH 7.4). Brains were then rapidly removed and postfixed at 4°C overnight. The medulla oblongata at the level of obex was sectioned coronally at a thickness of 10 μm. Sections were then incubated with a mouse anti-nNOS antiserum (1:25; Santa Cruz Biotechnology, Santa Cruz, CA USA) and a rabbit anti-PKA antiserum (1:50; Santa Cruz Biotechnology) for 24 h at 4°C followed by 1-h incubation of Alexa Fluor 546-conjugated goat anti-mouse IgG (1:125; Invitrogen, CA, USA) and Alexa Fluor 488-conjugated donkey anti-rabbit IgG (1:250; Invitrogen). Nuclear staining was performed with 4'-6-diamidino-2-phenylindole (DAPI) (1:250; Invitrogen, Carlsbad, CA, USA) in PBS for 10 min at room temperature. Immunoreactive expression of proteins was observed with a laser confocal microscope (Leica, Wetzlar, Germany).

### Western blotting

The experimental protocols for Western blot analysis of ADM-induced protein expression were described previously [12]. In brief, tissues from separate groups of rats obtained 10, 30 or 60 min after bilateral microinjections of aCSF or test agents into NTS were collected. The tissues covering the anatomical boundaries of the dorsomedial NTS were visualized and micropunched with the aid of a dissecting microscope. After tissue homogenization and protein quantification, proteins of interest were separated using a 12% SDS-polyacrylamide gel and transferred to a polyvinylidene difluoride (PVDF) membrane. Following blocking of non-specific binding, membranes were incubated with a rabbit anti-PKG-1α antiserum (1:2000; Calbiochem/EMD Biosciences, Darmstadt, Germany), a rabbit anti-nNOS antiserum (1:1000; Santa Cruz Biotechnology), a rabbit anti-phospho-nNOS (Ser847) antiserum (1:3000; Abcam, Cambridge, UK), a rabbit anti-phospho-nNOS (Ser1416) antiserum (1:3000; Abcam), or a rabbit anti-β-actin antiserum (1:10000; Santa Cruz Biotechnology) in Tris buffer at 4°C overnight. This was followed by incubation with horseradish peroxidase (HRP)-conjugated donkey anti-rabbit IgG (1:10000; Santa Cruz Biotechnology) for 1 h at room temperature. Western blots were quantified by densitometer and the relative density of proteins of interest was normalized against β-actin.

### Statistical analysis

All data are presented as mean ± S.E.M. Results were analyzed by one-way or two-way ANOVA with repeated measures for group means, as appropriate, followed by Scheffe's post hoc test for individual means. *P *< 0.05 was taken as statistically significant.

## Results

### Involvement of nNOS in BRR-enhancing effect of ADM in NTS

**Figure 1:**
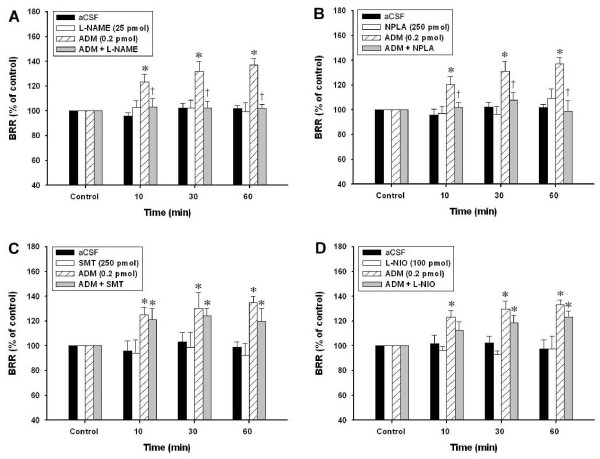
**Involvement of nNOS-dependent mechanism in the effect of ADM on BRR response**. A: Temporal changes in BRR response of the rat that received bilateral microinjections into NTS of aCSF, L-NAME (25 pmol), ADM (0.2 pmol) or ADM plus L-NAME. B: Temporal changes in BRR response of the rat that received bilateral microinjections into NTS of aCSF, NPLA (250 pmol), ADM (0.2 pmol) or ADM plus NPLA. C: Temporal changes in BRR response of the rat that received bilateral microinjections into NTS of aCSF, SMT (250 pmol), ADM (0.2 pmol) or ADM plus SMT. D: Temporal changes in BRR response of the rat that received bilateral microinjections into NTS of aCSF, L-NIO (100 pmol), ADM (0.2 pmol) or ADM plus L-NIO. Data are presented as means ± SEM, n = 6 to 8 animals per group. * p < 0.05 compared with control; † p < 0.05 compared with the ADM group at 10, 30 or 60 mins.

In our previous study [12], we have demonstrated that microinjection of ADM (0.2 pmol) into NTS significantly augmented BRR response with a maximal enhancement at 60 min after administration. Our first set of experiments established the participation of nNOS in this process. Microinjection bilaterally of ADM (0.2 pmol) into NTS elicited a 1.4 fold increase in the BRR response (Figure 1A). L-NAME (25 pmol) blunted completely the BRR-augmenting effect of ADM (0.2 pmol) when co-microinjected with ADM (Figure 1A). However, L-NAME, when given alone to NTS at 25 pmol, exerted minimal effect on BRR response (Figure 1A). Comparable effects were obtained on co-microinjection of ADM with NPLA (250 pmol), a selective nNOS inihibitor (Figure 1B). On the other hand, the ADM-induced BRR enhancement was substantially unaffected by co-microinjections with the relatively selective inducible NOS (iNOS) inhibitor SMT (250 pmol) (Figure 1C), or L-NIO (100 pmol), a preferential endothelial NOS (eNOS) inhibitor (Figure 1D).

### nNOS-dependent PKG activation by ADM and in NTS

**Figure 2:**
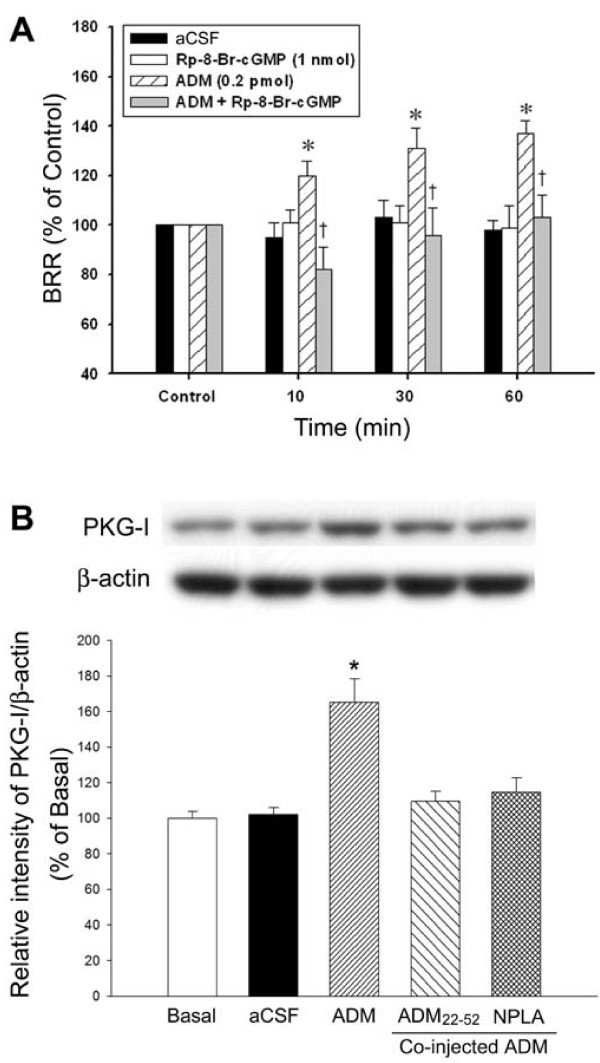
**Involvement of cGMP-dependent mechanism in the effect of ADM on BRR response**. A: Temporal changes in BRR response of the rat that received bilateral microinjections into NTS of aCSF, Rp-8-Br-cGMP (1 nmol), ADM (0.2 pmol) or ADM plus Rp-8-Br-cAMP. Data are presented as means ± SEM; *p < 0.05 compared with control; † p < 0.05 compared with the ADM group at 10, 30 or 60 mins. B: Representative gels (inset) and quantified data showing changes in the protein level of active form PKG-I in NTS of the rat at 30 min after receiving bilateral microinjections into NTS of aCSF, ADM (0.2 pmol), ADM plus ADM_22-52 _(0.2 pmol), or ADM plus NPLA (250 pmol). Quantified data are presented as means ± SEM. The mean value of sham-operated control rats is represented as Basal. * p < 0.05 compared with aCSF.

Since PKG can be activated by nNOS-derived NO [18], we next examined the role of PKG in the BRR enhancement response induced by ADM in NTS. Figure 2A shows that co-microinjection of Rp-8-Br-cGMP (1 nmol), a selective PKG inhibitor, abolished the ADM-elicited BRR augmentation. Western blot analysis revealed that ADM significantly increased PKG-I level in NTS 30 min after application, and was diminished by the ADMR antagonist ADM_22-52 _or NPLA (Figure 2B).

### Phosphorylation of nNOS by ADM in NTS

**Figure 3:**
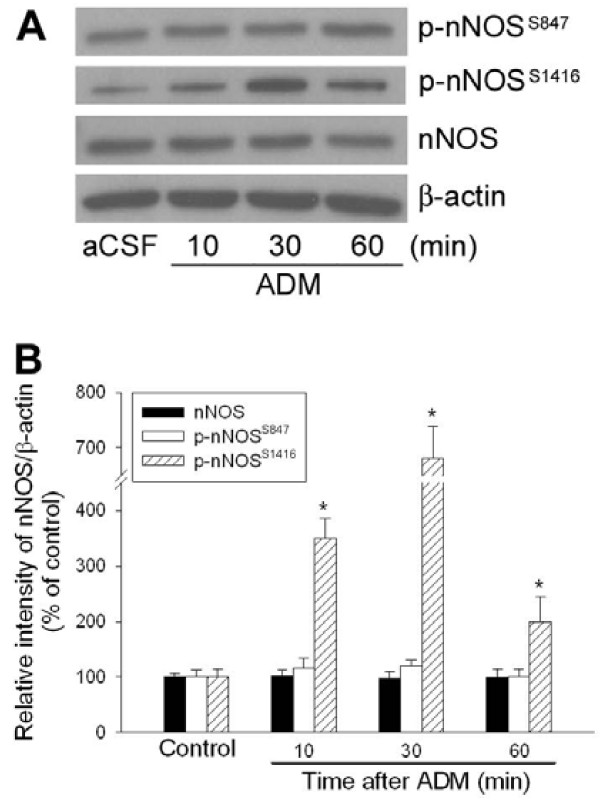
**ADM-induced increase in phosphorylation of nNOS**. A: Representative Western blotting gels showing temporal changes in phosphorylation at Ser847 (p-nNOS^S847^) and Ser1416 of nNOS (p-nNOS^S1416^) at 10, 30, and 60 min after intra-NTS microinjection of ADM (0.2 pmol). B: Quantified data showing temporal changes in the protein level of phospho-nNOS in NTS of the rat that received bilateral microinjections into NTS of aCSF (Control; sampled at 10 min after aCSF administration), or ADM (0.2 pmol). Data are presented as means ± SEM. * p < 0.05 compared with control.

Phosphorylation at critical amino acid residues is important for the regulation of nNOS activity [19]. Since ADM induces dephosphorylation of nNOS at Ser847 and stimulates NO production from cultured hypothalamic neurons [20], we examined the effect of ADM on phosphorylation of nNOS at Ser847. As shown in Figure 3A, the protein level of total nNOS was not substantially changed 10, 30, and 60 min after ADM administration. We also found that the protein levels of phospho-nNOS (Ser847) were not significantly altered during the time-period when BRR response was augmented by ADM (Figure 3A &3B).

In NTS, insulin-mediated cardiovascular effect was reported to involve the nNOS activation via phosphorylation at Ser1416 [21]. As illustrated in Figure 3A, ADM induced a significant increase in protein levels of phospho-nNOS (Ser1416) in NTS. The ADM-induced increase in phosphorylation of nNOS at Ser1416 maximized at 30 min and gradually declined within 60 min after ADM administration (Figure 3B).

### PKA-dependent activation of nNOS induced by ADM in NTS

**Figure 4:**
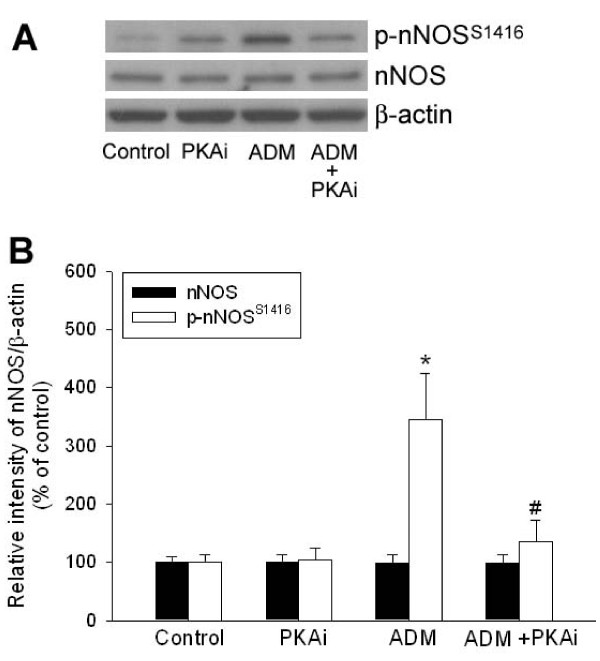
**ADM-induced PKA-dependent phosphorylation of nNOS**. Representative Western blotting gels showing changes in phosphorylation at Ser1416 of nNOS (p-nNOS^S1416^) at 10 min after intra-NTS microinjection of aCSF (control), PKA inhibitor Rp-8-Br-cAMP (PKAi; 1 nmol), ADM (0.2 pmol), or ADM plus PKAi. B: Quantified data showing changes in the protein level of phospho-nNOS in NTS of the rat that received bilateral microinjections into NTS of aCSF, PKAi (1 nmol), ADM (0.2 pmol), or ADM plus PKAi. Data are presented as means ± SEM. *p < 0.05 compared with control; #p < 0.05 compared with the ADM group.

In addition to the nNOS/PKG pathway, we demonstrated previously that the cAMP/PKA mechanism mediates the effects of ADM on baroreflex in NTS in rats [12]. Since both PKA [12] and nNOS (Figure 1) inhibitors abolished completely the ADM-elicited augmentation of BRR response, it is plausible that an in-series relationship exists between PKA and nNOS signaling pathways in the mediation of ADM effects in NTS. Our fourth series of experiments was carried out to examine whether nNOS phosphorylation is dependent on PKA activation evoked by ADM in NTS. We found that ADM-induced increase in phospho-nNOS (Ser1416) level was completely suppressed by co-microinjection with the PKA inhibitor Rp-8-Br-cAMP into NTS, while the level of total nNOS remained unaltered (Figure 4).

**Figure 5:**
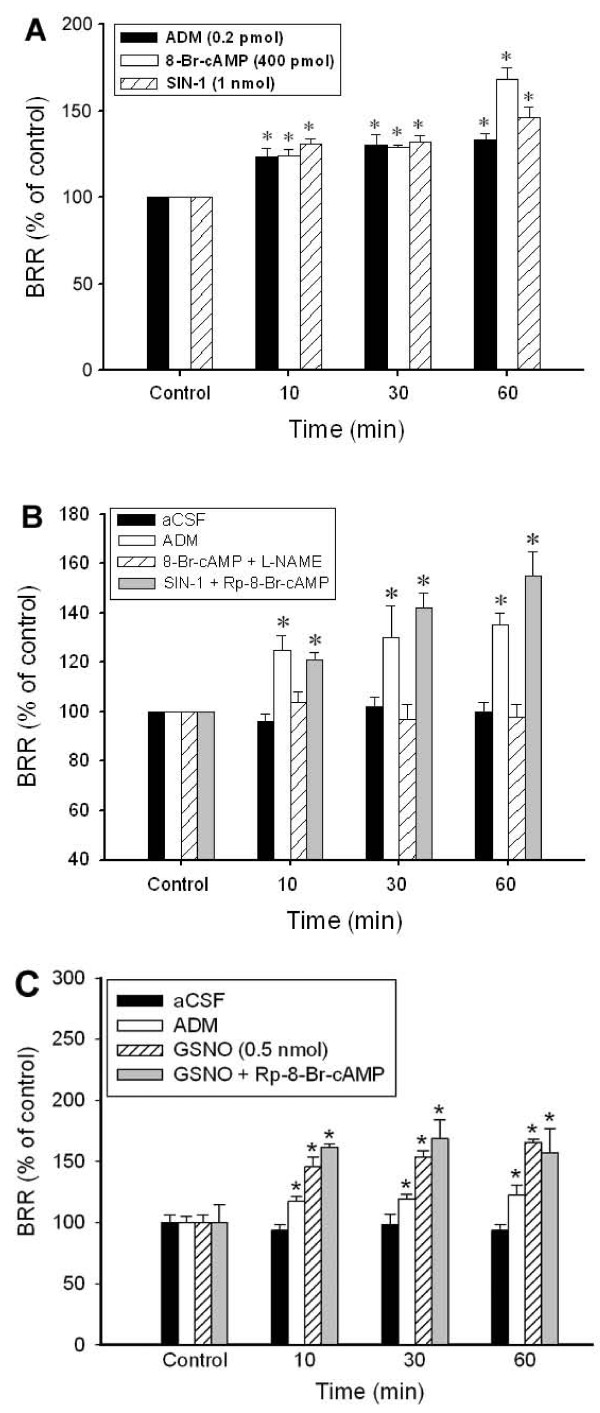
**Involvement of PKA-dependent NOS activation in the effect of ADM on BRR response**. A: Temporal changes in BRR response of the rat that received bilateral microinjections into NTS of ADM (0.2 pmol), 8-Br-cAMP (400 pmol), or SIN-1 (1 nmol). B: Temporal changes in BRR response of the rat that received bilateral microinjections into NTS of aCSF, ADM (0.2 pmol), 8-Br-cAMP (400 pmol) plus L-NAME (25 pmol), or SIN-1 (1 nmol) plus Rp-8-Br-cAMP (1 nmol). C: Temporal changes in BRR response of the rat that received bilateral microinjections into NTS of aCSF, ADM (0.2 pmol), GSNO (0.5 nmol), or GSNO plus Rp-8-Br-cAMP (1 nmol). Data are presented as means ± SEM, n = 6 to 8 animals per group. * p < 0.05 compared with control at 10, 30 or 60 mins.

We then verified the contribution of PKA-mediated nNOS activation to BRR augmentation. As illustrated in Figure 5A, both 8-Br-cAMP (400 pmol), a PKA activator, and SIN-1 (1 nmol), a putative NO donor, mimicked the BRR-enhancing effect of 0.2 pmol ADM at 10-60 min after microinjection into NTS. We further found that the BRR enhancement induced by 8-Br-cAMP was completely blocked by L-NAME (Figure 5B). On the other hand, the BRR-enhancing effect of SIN-1 was not altered by the PKA inhibitor Rp-8-Br-cAMP (Figure 5B). Of note is that the BRR augmentation by microinjection of GSNO (0.5 nmol), a specific NO donor, was comparable to that of SIN-1 and was also unaffected by co-microinjection with Rp-8-Br-cAMP (Figure 5C).

**Figure 6:**
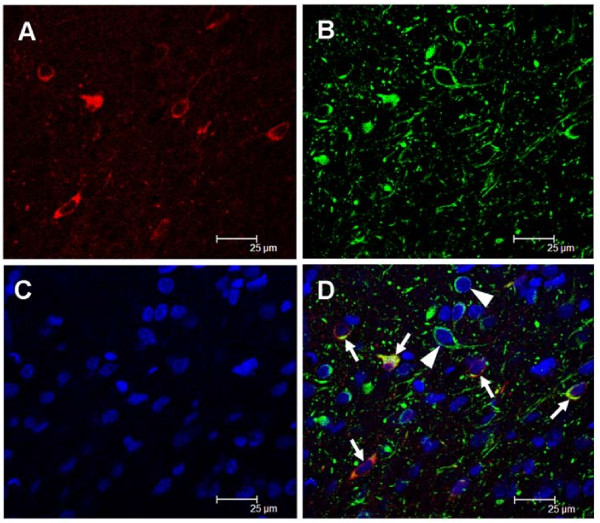
**Co-localization of PKA and nNOS proteins in NTS**. Confocal microscopic images of NTS showing immunofluorescence staining for nNOS (A, Alexa Fluor 546), PKA (B, Alexa Fluor 488), or cell nuclei (C, DAPI). The merged image (D) showing single staining for PKA (arrowhead) or double immunofluorescence staining (yellow color) for PKA and nNOS (arrows). Scale bar: 25 μm.

To determine whether nNOS and PKA are co-localized at the same NTS neuron, double immunohistochemical staining for nNOS and PKA proteins was carried out in rat brain slices. As shown in Figure 6, putative NTS neurons positively expressed nNOS-immunoreactivity (IR) were also stained with immunofluorescence for PKA protein, while some neurons manifested PKA-IR alone.

## Discussion

**Figure 7:**
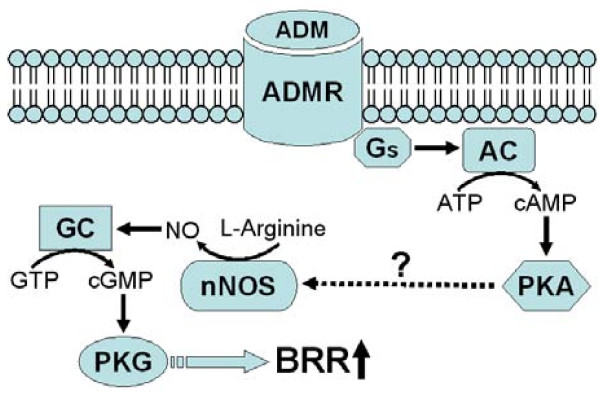
**Schematic model of cellular mechanisms underlying the enhancement of BRR response by ADM in NTS**. AC: adenylate cyclase; ADM: adrenomedullin; ADMR: adrenomedullin receptor; GC: guanylate cyclase; Gs: stimulatory GTP-binding protein; nNOS: neuronal nitric oxide synthase; PKA: protein kinase A; PKG: protein kinase G.

The present study unveiled two novel findings. We found that the activation of nNOS/PKG cascade is responsible for BRR enhancement induced by microinjection of ADM into the NTS. We further showed that activation of nNOS by ADM is via a PKA-dependent mechanism. Together with our previous findings [12], this study demonstrated that the signal transduction cascade that underlies the enhancement of BRR response by ADM in NTS is composed sequentially of cAMP/PKA and nNOS/PKG pathways (Figure 7).

This is the first report that provides direct biochemical and pharmacologic evidence to show that PKG-I, the active form of PKG, in NTS was upregulated by ADM-induced nNOS activation. NO participates in a wide variety of neuronal functions in the CNS, including cardiovascular regulation, nociception, synaptic plasticity, and control of complex behavioral responses (for review see [22]). At the NTS level, NO has been suggested to affect neuronal discharge and modulate the BRR response of the rat [23-26]. Although all three NOS isoforms have been suggested to be presented in the NTS [27,28], the possibility that the activation of iNOS and eNOS may be involved in ADM-induced BRR-enhancing effect is deemed unlikely (Figure 1C &1D). Moreover, several lines of evidence support the notion that nNOS-derived NO in NTS plays important physiologic roles in regulating transmission of arterial baroreflex signals and cardiovascular functions [29,30]. We further demonstrated in this study that the BRR-enhancing effect of ADM is mediated by nNOS-dependent PKG activation in NTS. We noted that the ADM-induced increase in nNOS phosphorylation declined gradually at 60 min after ADM microinjection, while the BRR-enhancing effect was sustained at the comparable time period. The discrepancy of temporal changes in nNOS activity and BRR response may reflect the sequential participation of nNOS and its downstream molecules including PKG in the ADM-activated signaling cascades. The significance of nNOS-dependent PKG activation in BRR regulation is further substantiated by a previous study [18] that revealed a significant nNOS-dependent upregulation of PKG-I protein in NTS following baroreceptor activation.

Another novel finding of the present study is that ADM may induce a PKA-dependent nNOS activation in NTS to enhance the BRR response. Both cAMP/PKA and NO/PKG mechanisms contribute to cardiovascular regulations by ADM in RVLM [11,16]. We further demonstrated these two signaling pathways exist in in-series in NTS. Our immunohistochemical results also showed that the PKA- and nNOS-dependent mechanisms could be activated in the same NTS neuron. We recognized that some PKA-labled NTS neurons did not expressed nNOS signal (Figure 6D). Recently, the extracellular signal-regulated kinase (ERK)-dependent signaling pathway in the NTS has been demonstrated to modulate cardiovascular functions [31]. It is thus possible that the PKA-dependent ERK signaling, which could be found in adipocytes activated by ADM [32], may serve as the downstream mechanism responsible for ADM-induced PKA activation in those NTS neurons expressing PKA-immunoreactivity only. The nNOS-IR is localized in neurons other than in glial cells [27] and is highly co-localized with soluble guanylate cyclase in NTS [33]. These results further substantiate our observations that ADM-activated nNOS/NO-cGMP/PKG cascades could be resided in the same NTS neuron.

The enzyme activity of nNOS has been demonstrated to be intimately associated with the state of phosphorylation at the amino acid residues Ser847 and Ser1416 [19]. For instance, phosphorylation of nNOS at Ser847 by calmodulin-dependent kinases results in a decrease of its enzyme activity [34]. On the other hand, protein phosphatase 2A-mediated dephosphorylation at Ser847 can lead to the activation of nNOS [35]. Recently, Xu and Krukoff demonstrated in an *in vitro *study that ADM significantly stimulated NO production from primary rat hypothalamic neurons by dephosphorylation of nNOS at Ser847 through a mechanism of PKA-dependent activation of phosphatases [20]. However, our results demonstrated that ADM induced an increase in phosphorylation of nNOS at Ser1416 but not at Ser847 in NTS neurons. The time course of nNOS phosphorylation is also compatible with the BRR-enhancing response induced by ADM. Since phosphorylation of nNOS at Ser1416, a known phosphorylation site for Akt (protein kinase B), is an alternative way to increase its enzyme activity [21], it is possible that Akt signaling may be involved in PKA-dependent nNOS phosphorylation and contributed to the ADM-induced BRR enhancement in NTS. This possibility, however, is subjected to further delineation.

## Conclusions

We have previously demonstrated an important role for ADM in BRR enhancement that is mediated by a PKA-dependent mechanism in the NTS [12]. In the present study, the effect of ADM on baroreflex was further suggested to involve the activation of nNOS in NTS. We conclude that the signal transduction cascade that underlies the enhancement of BRR response by ADM in NTS is composed sequentially of cAMP/PKA and nNOS/PKG pathways. These findings may provide a new insight for our understanding of ADM-elicited signaling mechanisms and their cross-talk in central regulation of cardiovascular functions.

## Competing interests

The authors declare that they have no competing interests.

## Authors' contributions

DHTY and LCC participated in the design of this study and helped to draft the manuscript. YCS carried out the immunohistochemical experiments. YCC and ICH carried out the neurophysiologic and neuropharmacologic studies, and performed the Western blotting analysis. YJL and ICC participated in the interpretation of data and performed the statistical analysis. JCY conceived of the study, designed and coordinated the experiments, and drafted the manuscript. All authors read and approved the final manuscript.

## References

[B1] Kitamura K Kangawa K Kawamoto M Ichiki Y Nakamura S Matsuo H Eto T Adrenomedullin - a novel hypotensive peptide isolated from human pheochromocytoma Biochem Biophys Res Commun 1993 192 553 560 10.1006/bbrc.1993.1451 8387282

[B2] Gibbons C Dackor R Dunworth W Fritz-Six K Caron KM Receptor activity-modifying proteins: RAMPing up adrenomedullin signaling Mol Endocrinol 2007 21 783 796 17053041 10.1210/me.2006-0156

[B3] Kuwasako K Kitamura K Ito K Uemura T Yanagita Y Kato J Sakata T Eto T The seven amino acids of human RAMP2 and RAMP3 are critical for agonist binding to human adrenomedullin receptors J Biol Chem 2001 276 49459 49465 10.1074/jbc.M108369200 11591721

[B4] Hwang IS Tang F The distribution and gene expression of adrenomedullin in the rat brain: peptide/mRNA and precursor/active peptide relationships Neurosci Lett 1999 267 85 88 10.1016/S0304-3940(99)00320-1 10400218

[B5] Oliver KR Kane SA Salvatore CA Mallee JJ Kinsey AM Koblan KS Keyvan-Fouladi N Heavens RP Wainwright A Jacobson M Dickerson IM Hill RG Cloning, characterization and central nervous system distribution of receptor activity modifying proteins in the rat Eur J Neurosci 2001 14 618 628 10.1046/j.0953-816x.2001.01688.x 11556887

[B6] Serrano J Uttenthal LO Martínez A Fernández AP Martínez de Velasco J Alonso D Bentura ML Santacana M Gallardo JR Martínez-Murillo R Cuttitta F Rodrigo J Distribution of adrenomedullin-like immunoreactivity in the rat central nervous system by light and electron microscopy Brain Res 2000 853 245 268 10.1016/S0006-8993(99)02273-8 10640622

[B7] Takahashi H Watanabe TX Nishimura M Nakanishi T Sakamoto M Yoshimura M Komiyama Y Masuda M Murakami T Centrally induced vasopressor and sympathetic responses to a novel endogenous peptide, adrenomedullin, in anesthetized rats Am J Hypertens 1994 7 478 482 8060585 10.1093/ajh/7.5.478

[B8] Taylor MM Samson WK Adrenomedullin and central cardiovascular regulation Peptides 2001 22 1803 1807 10.1016/S0196-9781(01)00522-8 11754966

[B9] Matsumura K Abe I Tsuchihashi T Fujishima M Central adrenomedullin augments the baroreceptor reflex in concious rabbits Hypertension 1999 33 992 997 10205236 10.1161/01.hyp.33.4.992

[B10] Taylor MM Keown CA Samson WK Involvement of the central adrenomedullin peptides in the baroreflex Regul Pept 2003 112 87 93 10.1016/S0167-0115(03)00026-0 12667629

[B11] Xu Y Krukoff TL Adrenomedullin in the rostral ventrolateral medulla inhibits baroreflex control of heart rate: a role for protein kinase A Br J Pharmacol 2006 148 70 77 16501581 10.1038/sj.bjp.0706698 PMC1617038

[B12] Ho LK Chen K Ho IC Shen YC Yen DHT Li FCH Lin YC Kuo WK Lou YJ Yen JC Adrenomedullin enhances baroreceptor reflex response via cAMP/PKA signaling in nucleus tractus solitarii of rats Neuropharmacology 2008 55 729 736 10.1016/j.neuropharm.2008.06.024 18616957

[B13] Spyer KM Neural organization and control of the baroreceptor reflex Rev Physiol Biochem Pharmacol 1981 88 23 124 10.1007/BFb0034536 7010509

[B14] Ishizaka Y Ishizaka Y Tanaka M Kitamura K Kangawa K Minamino N Matsuo H Eto T Adrenomedullin stimulates cyclic AMP formation in rat vascular smooth muscle cells Biochem Biophys Res Commun 1994 200 642 646 10.1006/bbrc.1994.1496 8166740

[B15] Geambasu A Krukoff TL Adrenomedullin acts in the parabrachial nucleus to increase arterial blood pressure through mechanisms mediated by glutamate and nitric oxide Am J Physiol 2008 295 R38 R44 10.1152/ajpcell.00548.2007 18495835

[B16] Xu Y Krukoff TL Adrenomedullin in the rostral ventrolateral medulla increases arterial pressure and heart rate: roles of glutamate and nitric oxide Am J Physiol 2004 287 R729 R734 10.1152/ajpregu.00188.2004 PMC4820402 15178541

[B17] Yang CCH Kuo TBJ Chan SHH Auto- and cross-spectral analysis of cardiovascular fluctuations during pentobarbital anesthesia in the rat Am J Physiol 1996 270 H575 H582 8779833 10.1152/ajpheart.1996.270.2.H575

[B18] Chan SHH Chang KF Ou CC Chan JYH Nitric oxide regulates c-fos expression in nucleus tractus solitarii induced by baroreceptor activation via cGMP-dependent protein kinase and cAMP response element-binding protein phosphorylation Mol Pharmacol 2004 65 319 325 10.1124/mol.65.2.319 14742673

[B19] Zhou L Zhu DY Neuronal nitric oxide synthase: structure, subcellular localization, regulation, and clinical implications Nitric Oxide 2009 20 223 230 10.1016/j.niox.2009.03.001 19298861

[B20] Xu Y Krukoff TL Adrenomedullin stimulates nitric oxide production from primary rat hypothalamic neurons Mol Pharmacol 2007 72 112 120 10.1124/mol.106.033761 17446268

[B21] Chiang HT Cheng WH Lu PJ Huang HN Lo WC Tseng YC Wang JL Hsiao M Tseng CJ Neuronal nitric oxide synthase activation is involved in insulin-mediated cardiovascular effects in the nucleus tractus solitarii of rats Neuroscience 2009 159 727 734 10.1016/j.neuroscience.2008.12.048 19167463

[B22] Prast H Philippu A Nitric oxide as modulator of neuronal functions Prog Neurobiol 2001 64 51 68 10.1016/S0301-0082(00)00044-7 11250062

[B23] Dias ACR Vitela M Colombari E Mifflin SW Nitric oxide modulation of glutamatergic, baroreflex, and cardiopulmonary transmission in the nucleus of the solitary tract Am J Physiol 2005 288 H256 H262 10.1152/ajpheart.01149.2003 15598868

[B24] Kong SZ Fan MX Zhang BH Wang ZY Wang Y Nitric oxide inhibits excitatory vagal afferent input to nucleus tractus solitarius neurons in anaesthetized rats Neurosci Bull 2009 25 325 334 10.1007/s12264-009-0624-x 19927168 PMC5552507

[B25] Lo WJ Liu HW Lin HC Ger LP Tung CS Tseng CJ Modulatory effects of nitric oxide on baroreflex activation in the brainstem nuclei of rats Chin J Physiol 1996 39 57 62 8902305

[B26] Pontieri V Venezuela MK Scavone C Michelini LC Role of endogenous nitric oxide in the nucleus tractus solitarii on baroreflex control of heart rate in spontaneously hypertensive rats J Hypertens 1998 16 1993 1999 10.1097/00004872-199816121-00021 9886888

[B27] Lin LH Taktakishvili O Talman WT Identification and localization of cell types that express endothelial and neuronal nitric oxide synthase in the nucleus tractus solitarii Brain Res 2007 1171 42 51 17761150 10.1016/j.brainres.2007.07.057 PMC2141649

[B28] Tai MH Weng WT Lo WC Chan JY Lin CJ Lam HC Tseng CJ Role of nitric oxide in alpha-melanocyte-stimulating hormone-induced hypotension in the nucleus tractus solitarii of the spontaneously hypertensive rats J Pharmacol Exp Ther 2007 321 455 461 10.1124/jpet.106.118299 17283224

[B29] Lin HC Wan FJ Tseng CJ Modulation of cardiovascular effects produced by nitric oxide and ionotropic glutamate receptor interaction in the nucleus tractus solitarii of rats Neuropharmacology 1999 38 935 941 10.1016/S0028-3908(99)00017-9 10428412

[B30] Talman WT Dragon DN Transmission of arterial baroreflex signals depends on neuronal nitric oxide synthase Hypertension 2004 43 820 824 10.1161/01.HYP.0000120848.76987.ef 14981065

[B31] Cheng WH Lu PJ Ho WY Tung CS Cheng PW Hsiao M Tseng CJ Angiotensin II inhibits neuronal nitric oxide synthase activation through the ERK1/2-RSK signaling pathway to modulate central control of blood pressure Circ Res 2010 106 788 795 10.1161/CIRCRESAHA.109.208439 20056918

[B32] Iemura-Inaba C Nishikimi T Akimoto K Yoshihara F Minamino N Matsuoka H Role of adrenomedullin system in lipid metabolism and its signalling mechanism in cultured adipocytes Am J Physiol Regul Integr Comp Physiol 2008 295 R1376 1384 10.1152/ajpregu.90467.2008 18685068

[B33] Lin LH Talman WT Soluble guanylate cyclase and neuronal nitric oxide synthase colocalize in rat nucleus tractus solitarii J Chem Neuroanat 2005 29 127 136 10.1016/j.jchemneu.2004.10.002 15652699

[B34] Hayashi Y Nishio M Naito Y Yokokura H Nimura Y Hidaka H Watanabe Y Regulation of neuronal nitric-oxide synthase by calmodulin kinases J Biol Chem 1999 274 20597 20602 10.1074/jbc.274.29.20597 10400690

[B35] Komeima K Watanabe Y Dephosphorylation of nNOS at Ser847 by protein phosphatase 2A FEBS Lett 2001 497 65 66 10.1016/S0014-5793(01)02389-4 11376664

